# Increased circulating concentrations of mesencephalic astrocyte-derived neurotrophic factor in children with type 1 diabetes

**DOI:** 10.1038/srep29058

**Published:** 2016-06-30

**Authors:** Emilia Galli, Taina Härkönen, Markus T. Sainio, Mart Ustav, Urve Toots, Arto Urtti, Marjo Yliperttula, Maria Lindahl, Mikael Knip, Mart Saarma, Päivi Lindholm

**Affiliations:** 1 Institute of Biotechnology, University of Helsinki, Finland; 2 Division of Pharmaceutical Biosciences, Centre for Drug Research, University of Helsinki, Finland; 3 Children’s Hospital, University of Helsinki and Helsinki University Central Hospital, Finland; 4 Research Programs Unit, Diabetes and Obesity, University of Helsinki, Finland; 5 Icosagen Ltd., Tartu, Estonia; 6 Folkhälsan Research Center, Helsinki, Finland

## Abstract

Mesencephalic astrocyte-derived neurotrophic factor (MANF) was recently shown to be essential for the survival and proliferation of pancreatic β-cells in mice, where deletion of MANF resulted in diabetes. The current study aimed at determining whether the concentration of circulating MANF is associated with the clinical manifestation of human type 1 diabetes (T1D). MANF expression in T1D or MANF levels in serum have not been previously studied. We developed an enzyme-linked immunosorbent assay (ELISA) for MANF and measured serum MANF concentrations from 186 newly diagnosed children and adolescents and 20 adults with longer-term T1D alongside with age-matched controls. In healthy controls the mean serum MANF concentration was 7.0 ng/ml. High MANF concentrations were found in children 1–9 years of age close to the diagnosis of T1D. The increased MANF concentrations were not associated with diabetes-predictive autoantibodies and autoantibodies against MANF were extremely rare. Patients with conspicuously high MANF serum concentrations had lower C-peptide levels compared to patients with moderate MANF concentrations. Our data indicate that increased MANF concentrations in serum are associated with the clinical manifestation of T1D in children, but the exact mechanism behind the increase remains elusive.

Type 1 diabetes is an autoimmune disorder characterized by selective loss of insulin producing β-cells in the pancreas^1^. The first signs of β-cell autoimmunity and compromised β-cell function can be observed months to years before the clinical disease presentation. Once a critical mass of functional β-cells is destroyed, the occurrence of symptoms, caused by lack of insulin, is acute. Differently from T1D, type 2 diabetes (T2D) is a metabolic disorder related to obesity and characterized by insulin resistance i.e., decreased action of insulin in muscle, liver and adipose tissue, and failure of β-cells to maintain sufficient insulin levels^2^. Interestingly, insulin secretion from adipocytes was recently reported suggesting a crosstalk between adipose tissue and pancreas in the regulation of energy metabolism in obesity and development of T2D^3^.

MANF (also known as arginine-rich, mutated in early stage tumors; ARMET)^4^ and the homologous cerebral dopamine neurotrophic factor (CDNF)^5^ form a novel protein family with neuroprotective activities *in vivo*^6^,^7^,^8^,^9^. A recent discovery has linked MANF to the development of insulin-dependent diabetes in mice^10^. Complete and pancreas-specific deletion of MANF caused severe diabetes characterized by postnatal reduction of β-cell mass, due to their increased apoptosis and decreased proliferation, indicating that MANF is essential for the maintenance of β-cells. Importantly, MANF was able to specifically enhance the regeneration of adult mouse β-cells *in vivo*, denoting that MANF has therapeutic potential for the treatment of T1D, which currently lacks disease modifying therapy.

MANF is an endoplasmic reticulum (ER) stress responsive protein^11^ and involved in the maintenance of ER homeostasis, which is important especially for professional secretory cells. Pancreatic β-cells, which express MANF abundantly^10^, are susceptible to ER stress due to high insulin production^12^. MANF expression is increased in β-cells suffering from constant ER stress due to misfolded proinsulin^11^,^13^. On the other hand, ER stress related unfolded protein response (UPR) pathways are activated in the pancreatic islets of mice lacking MANF^10^. MANF improves cell viability under treatments causing ER stress *in vitro*^14^,^15^,^16^ and protects cortical neurons and cardiac myocytes against ischemia *in vivo*^8^,^17^, a known inducer of ER stress and UPR^18^. MANF is also a secreted protein^14^,^19^ and ER stress increases MANF secretion as shown *in vitro* in cell lines^15^,^17^,^20^ and *in vivo* in a mouse model of skeletal disease^21^.

MANF has recently been studied in relation to other human autoimmune diseases, namely rheumatoid arthritis and systemic lupus erythematosus, conditions also involving ER stress, where MANF expression was found increased in circulating leucocytes^22^,^23^. Furthermore, MANF is involved in the regulation of inflammation as recombinant MANF decreased the expression and secretion of cytokines under inflammatory conditions *in vitro*^16^,^23^.

Increasing data suggest that MANF is essential for the function and survival of β-cells. Development of MANF-based therapies for diabetes requires detailed characterization of MANF expression and activity. The current study aimed at determining whether serum MANF is associated with newly diagnosed T1D in humans. MANF concentrations in human serum, in health or disease, have not been previously determined.

## Results

### Development of specific ELISA for quantification of human MANF

**Figure 1:**
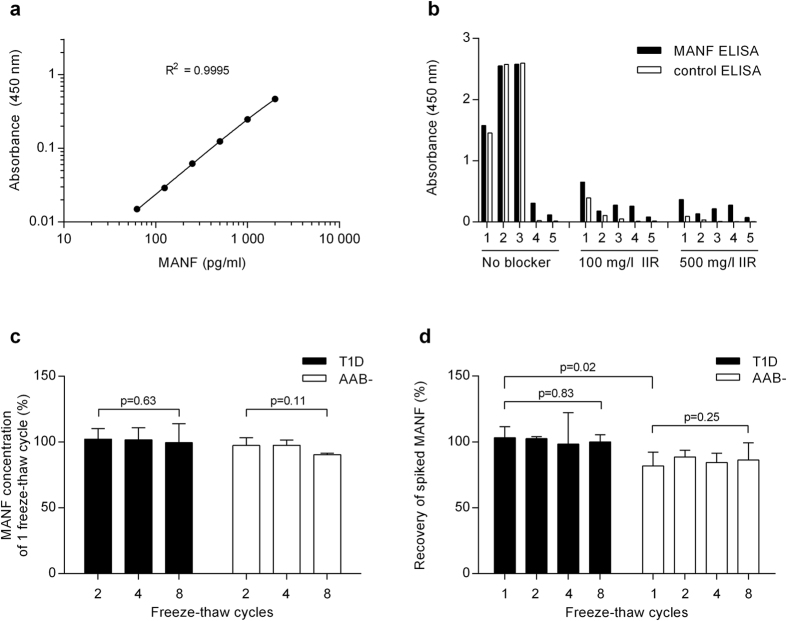
Optimization of the ELISA for measurement of MANF in human serum. (**a**) A typical standard curve of MANF ELISA on a log-log scale. (**b**) Analysis of assay interference by the control ELISA using samples with high (1–3) or low interference (4, 5). A background of 25%, 26%, 4%, 2% and 6% from the MANF ELISA readings remained for the samples 1–5, respectively, with the IIR concentration of 500 mg/l. (**c**) Stability of endogenous MANF in serum samples from type 1 diabetes patients (T1D) and autoantibody-negative controls (AAB−) under repeated freeze-thaw cycles. The results are presented as % from the MANF concentration measured in the sample of one freeze-thaw cycle. (**d**) Stability of spiked MANF (á 500 pg/ml) in serum samples under repeated freeze-thaw cycles. The results are presented as % recovery of 500 pg/ml spike after the concentration of endogenous MANF, analyzed in a parallel sample, was subtracted. Columns represent the average + SD of recoveries in 4 serum samples. Statistical significance was analyzed by repeated measures ANOVA between repeated freeze-thaw cycles [2, 4, and 8 for (**c**), and 1, 2, 4, and 8 for (**d**)], and by independent samples t-test between the two groups in case of 1 freeze-thaw cycle in (**d**).

A typical standard curve of the MANF ELISA is presented in Fig. 1a. Within the assay dynamic range of 62.5 to 2,000 pg/ml, MANF could be measured with acceptable accuracy and precision (Supplementary Table S1). Sensitivity of the assay was 45 pg/ml. Intra-assay and interassay precision values were within 4.4 to 11.5% Coefficient of Variation (CV) and 3.1 to 6.8% CV, respectively (Supplementary Table S2). The assay did not recognize recombinant human CDNF, or endogenous mouse MANF and CDNF present in testis and heart lysates^5^,^19^ (Supplementary Table S3).

Heterophilic antibodies present in serum can interfere with immunological assays^24^. Interference by serum heterophilic antibodies was evaluated by a control ELISA, which was run exactly as the MANF ELISA except that the coating antibody was goat anti-human CDNF instead of goat anti-human MANF. The control ELISA did not detect either recombinant human CDNF or MANF at a concentration of 2,000 pg/ml but gave positive results for human sera (Fig. 1b). Human tissue lysates, which gave a signal on MANF ELISA, were negative on the control ELISA (data not shown). By using an antibody pair originating from the same host species as the antibodies used in MANF ELISA, we consider that the control ELISA revealed all background caused by heterophilic antibodies present in human serum.

Addition of a commercial heterophilic antibody blocker, Immunoglobulin Inhibiting Reagent (IIR; Sera Lab, West Sussex, U.K.), to the serum samples reduced the background optical density (OD) values measured on the control ELISA more than the OD values measured on the MANF ELISA. The IIR concentration of 500 mg/l was more efficient in reducing the background compared to the concentration of 100 mg/l (Fig. 1b), and was used in the analysis. Recovery of spiked MANF and dilutional linearity of the serum samples treated with IIR were within a range of 93.5 to 115.5% (Supplementary Table S4). Remaining background in 32 (91%) out of 35 samples tested was maximally 11.3% of the OD values measured by the MANF ELISA (Supplementary Table S5).

### MANF stability in serum *in vitro*

Serum samples analyzed in the present study were stored at −80 °C and thawed before analysis. We tested the stability of MANF in human serum obtained from T1D patients or autoantibody-negative healthy controls after up to eight freeze-thaw cycles. Concentrations of endogenous MANF remained stable after repeated freeze-thaw cycles as detected by ELISA. After 8 freeze-thaw cycles 99.6 ± 14.3% and 90.4 ± 1.6% of endogenous MANF was detected in T1D (*n* = 4) and autoantibody-negative sera (*n* = 4), respectively, compared to the samples with only 1 freeze-thaw cycle (Fig. 1c). Recovery of spiked MANF after 1 freeze-thaw cycle was 103.1 ± 8.5% and 81.7 ± 10.5% in T1D (*n* = 4) and autoantibody-negative sera (*n* = 4), respectively (Fig. 1d). The recovery was statistically significantly lower from the autoantibody-negative sera compared to that from the T1D sera (*p* = 0.02). Stability of spiked MANF after 8 freeze-thaw cycles was 97.6 ± 10.4% and 105.2 ± 4.9% compared to MANF concentration in the samples with 1 freeze-thaw cycle in T1D (*n* = 4) and autoantibody-negative sera (*n* = 4), respectively (Fig. 1d).

### Stratification of the study population

The incidence of T1D shows classically a peak in puberty^25^, which is the time of hormonal and metabolic changes. At the diagnosis of T1D the lowest functional β-cell mass, estimated by serum C-peptide levels, is seen in the youngest patients whereas older subjects have a better preserved β-cell function at diagnosis^26^. Since the age at T1D onset affects the disease course we decided to stratify the study participants into three age groups based in the relation to puberty: prepubertal children age of 1–9 years, older pubertal children and adolescents age of 10–17 years, and adults age of 25–52 years. The characteristics of the study groups are presented in Table 1.

### Average serum MANF concentration in the non-diabetic control population

The average (median) concentration of circulating MANF in the healthy autoantibody-negative control population comprising children, adolescents and adults (*n* = 113) was 7.0 ± 3.1 (6.6) ng/ml (range 1.4 to 21.8 ng/ml). The mean age in the control population was 14.6 ± 12.0 years (range 1 to 50.9 years). The average concentration of serum MANF did not differ statistically significantly between the three age groups of 1–9-year-olds, 10–17-year-olds, and 25–52-year-olds among the autoantibody-negative study subjects (*p* = 0.22). Neither did we observe significant differences in serum MANF concentrations between females and males in any of the three age groups studied (*p* = 0.48, *p* = 0.35, and *p* = 0.88, for 1–9, 10–17, and 25–52-year-olds, respectively).

### Increased serum MANF concentrations in children at the onset of T1D

**Figure 2:**
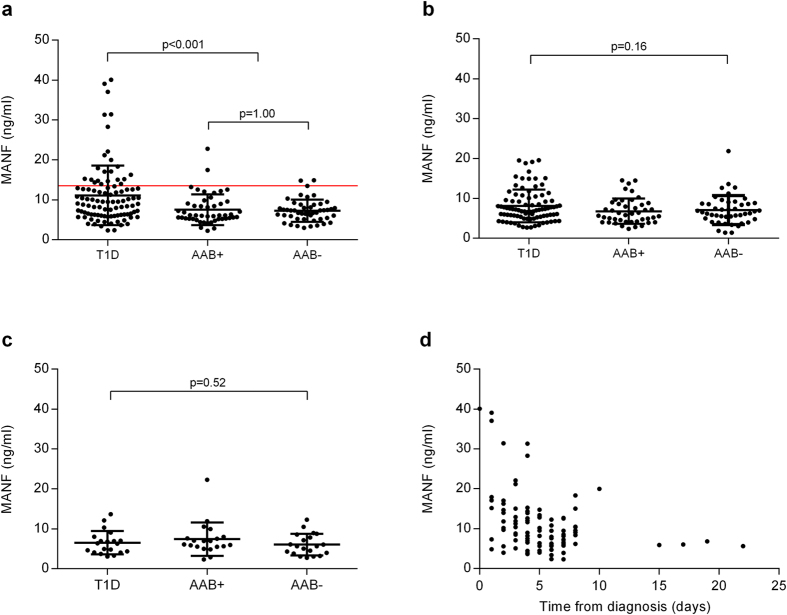
Increased MANF concentrations in children at the clinical manifestation of T1D. (**a**) Higher serum MANF concentrations in 1–9-year-old children with T1D (T1D, *n* = 98) compared to non-diabetic autoantibody-positive (AAB+, *n* = 48) and autoantibody-negative (AAB−, *n* = 48) controls. The red horizontal line indicates a cut-off limit for MANF (13.5 ng/ml), within which 95% of the controls fell. (**b**) MANF concentrations in 10–17-year-old T1D patients (*n* = 88) were comparable to the autoantibody-positive (*n* = 44) and autoantibody-negative (*n* = 45) controls. (**c**) MANF concentrations in 25–52-year-old adults with longer-term T1D did not differ from the controls (*n* = 20, for all). (**d**) Inverse correlation of serum MANF with the time taken from diagnosis to sample collection in 1–9-year-old T1D patients (r_s_ = −0.35, *p* < 0.001 *n* = 98). Every black dot represents one sample and the horizontal lines indicate the mean ± SD. Statistical significance was analyzed by Kruskall-Wallis H test in (**a–c**) in adjunct with Tukey HSD post-hoc test in (**a**), and by Spearman’s rank correlation (**d**).

MANF concentrations in serum samples derived from 1–9-year-old patients with recent onset T1D (*n* = 98, sample taken within 0–22 days from the clinical diagnosis) were analyzed along with two age-matched control groups, one comprising non-diabetic siblings testing positive for two to five of the diabetes-predictive autoantibodies analyzed (*n* = 48), and the other being autoantibody-negative (*n* = 48). The average (median) concentration of serum MANF in T1D, autoantibody-positive and autoantibody-negative group was 11.1 ± 7.5 (9.5), 7.5 ± 3.8 (6.1) and 7.2 ± 2.8 (7.0) ng/ml, respectively (Table 2; Fig. 2a). Patients with T1D had 3.9 ng/ml (54%) higher average serum MANF concentration compared to the autoantibody-negative control group (*p* < 0.001). The group of T1D patients differed statistically significantly also from the autoantibody-positive control group (*p* < 0.001). The autoantibody-positive and autoantibody-negative control groups were comparable to each other (*p* = 1.0). MANF concentration and the time from T1D diagnosis to sample collection correlated inversely in the group of 1–9-year-old patients (r_s_ = −0.35, *p* < 0.001, *n* = 98, Fig. 2d).

The average (median) serum MANF concentration in 10–17-year-old patients with recent onset T1D (sample taken 0–24 days from the diagnosis; *n* = 88) was 8.1 ± 4.1 (7.2) ng/ml, whereas in the autoantibody-positive (*n* = 44) and autoantibody-negative (*n* = 45) controls it was 6.8 ± 3.2 (6.0) ng/ml and 7.1 ± 3.7 (6.6) ng/ml, respectively (Table 2). The groups did not differ significantly from each other in terms of MANF concentration (*p* = 0.16; Fig. 2b).

In adult longer-term T1D patients (sample taken 3.8–40.3 years from the diagnosis, *n* = 20), and age matched autoantibody-positive (*n* = 20) and autoantibody-negative (*n* = 20) control subjects the average (median) concentration of serum MANF was 6.5 ± 2.9 (6.3), 7.4 ± 4.2 (6.5) and 6.0 ± 2.7 (5.6) ng/ml, respectively (Table 2). The average concentration of serum MANF did not differ between the groups (*p* = 0.52; Fig. 2c).

### Extremely high serum MANF concentrations in a subset of 1–9-year-old children with T1D

Within the group of 1–9-year-old children with T1D, the highest serum MANF concentration measured was 40 ng/ml (Fig. 2a). We wanted to characterize more closely the serum samples with very high MANF concentrations obtained from T1D children. Based on MANF concentrations measured from serum samples of the 1–9-year-old autoantibody-negative control subjects, we determined a 95% cut-off limit for serum MANF concentration. MANF serum concentration in 95% of the autoantibody-negative controls (*n* = 46/48) and also in 95% of autoantibody-positive controls (*n* = 46/48) was equal or below to that of 13.5 ng/ml. In the case of T1D patients, 23.5% (*n* = 23/98) had higher MANF serum concentration than 13.5 ng/ml (Fig. 2a, vertical line). We observed that the samples with a MANF concentration of more than 13.5 ng/ml had been taken closer to the clinical diagnosis of T1D (3.3 ± 2.5 days; *n* = 23) compared to the samples with lower MANF levels (5.8 ± 3.6 days, *n* = 75; *p* < 0.001). In addition, the patients with MANF concentration over 13.5 ng/ml were younger compared to the patients with lower MANF concentration (5.0 ± 2.5 vs. 6.3 ± 2.5 years of age, *p* = 0.027).

### Low C-peptide levels in children with high MANF concentration

To study whether the increase in serum MANF concentrations observed in children with newly diagnosed T1D is related to the amount of remaining functional β-cell mass, we analyzed serum C-peptide levels. C-peptide is secreted from the β-cells together with insulin in an equimolar proportion and its levels are considered as a measure of functional β-cell mass^26^. Average C-peptide concentrations in the study groups are presented in Table 3. There was no statistically significant difference between the average C-peptide levels of 1–9-year-old (*n* = 84) and 10–17-year-old (*n* = 79) T1D patients (*p* = 0.24). In adults with longer-term T1D (*n* = 20), C-peptide levels were significantly lower than in the 1–9-year-olds and the 10–17-year-olds (*p* < 0.001, for both).

**Figure 3:**
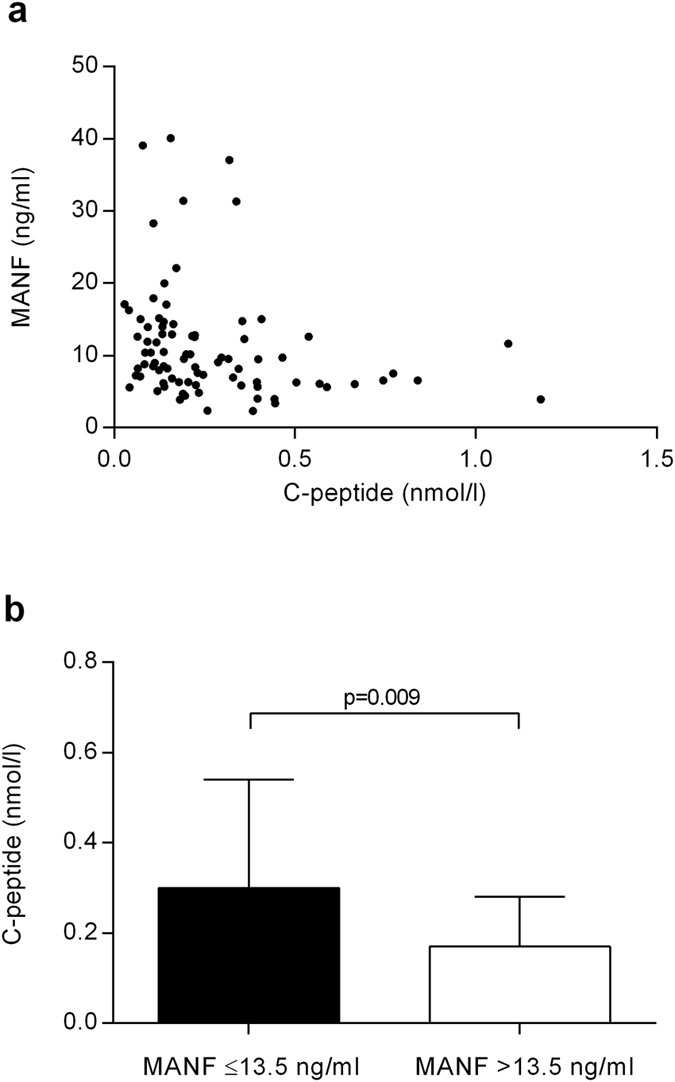
Lower C-peptide levels with higher MANF concentration. (**a**) Inverse correlation of MANF and C-peptide concentrations in the sera of 1–9-year-old children with T1D (r_s_ = −0.37, *p* = 0.001, *n* = 84). (**b**) Average C-peptide concentration was statistically significantly lower in 1–9-year-old children with extremely high MANF concentration (>13.5 ng/ml, *n* = 20) compared to the children with lower MANF concentration (≤13.5 ng/ml, *n* = 64) at the onset of T1D. Statistical significance was analyzed by Spearman’s rank correlation (**a**) and by Mann-Whitney U test (**b**).

In 1–9-year-old patients with T1D, C-peptide levels correlated inversely with MANF concentrations (r_s_ = −0.37, *p* = 0.001, *n* = 84, Fig. 3a). Both MANF (r_s_ = −0.38, *p* < 0.001, *n* = 84, Supplementary Fig. S1) and C-peptide (r_s_ = 0.36, *p* = 0.001, *n* = 84, Supplementary Fig. S2) correlated with the time (in days) from diagnosis to sample collection. Partial correlation, having time from diagnosis as the controlling factor for ranked MANF and C-peptide values, indicated that the correlation between MANF and C-peptide was not dependent on the time from diagnosis (r = −0.28, *p* = 0.009). In contrast to the group of 1–9-year-old patients with T1D, C-peptide and MANF levels did not correlate in the group of 10–17-year-old patients (r_s_ = −0.006, *p* = 0.96, *n* = 79).

Children 1–9 years of age with high (>13.5 ng/ml) serum MANF concentration had approximately 43% lower C-peptide levels compared to the samples with MANF concentration ≤13.5 ng/ml (0.17 ± 0.11 nmol/l, *n* = 20, vs. 0.30 ± 0.24 nmol/l, *n* = 64, *p* = 0.009, Fig. 3b).

### No associations between MANF concentrations and the presence of T1D-predictive autoantibodies

We analyzed the association of serum MANF concentration in the 1–9-year-old children with the occurrence of the five most prevalent and best characterized autoantibodies predictive of T1D, namely islet cell autoantibodies (ICA), insulin autoantibodies (IAA), and autoantibodies to glutamic acid decarboxylase (GADA), islet antigen 2 (IA-2A), and zinc transporter 8 (ZnT8A). Children with T1D tested positive for one to five autoantibodies, and autoantibody-positive subjects had two to five autoantibodies. We found no differences in the MANF serum concentration in relation to the presence of autoantibodies in patients with T1D (*n* = 51–95, depending on the autoantibody) or in non-diabetic autoantibody-positive children (*n* = 48; Supplementary Table S6). Furthermore, the total number of autoantibodies was not associated with serum MANF concentration in children with newly diagnosed T1D. The subset of 1–9-year-old children with MANF concentration over 13.5 ng/ml did not differ from the group of children with MANF concentrations equal or below 13.5 ng/ml in terms of autoantibody prevalence.

The occurrence of T1D-predictive antibodies was not associated with the average MANF concentration in the 10–17-year-old or 25–52-year-old T1D patients or autoantibody-positive controls (Supplementary Table S6).

### Autoantibodies to MANF are not detected in the serum of patients with T1D

Counts per minute (cpm) values of MANF immune complexes in the serum from children and adolescents with T1D (*n* = 94), autoantibody-positive (*n* = 32) and autoantibody-negative controls (*n* = 91) were very low. There was only one sample from a patient with diabetes, which repeatedly gave responses above the background [5 standard deviations (SDs) above the mean cpm value of autoantibody-negative controls; data not shown].

### Cytokine levels in children with extremely high serum MANF concentrations

Since MANF has been associated with regulation of cytokine expression, we set out to study whether high concentrations of serum MANF in 1–9-year-old children with T1D would be related to circulating cytokine levels. We analyzed the concentration of interleukin (IL)-1β, IL-2, IL-4, IL-5, IL-6, IL-10, IL-12 (p70), IL-13, interferon (IFN)-γ and tumor necrosis factor (TNF)-α in the serum samples from T1D patients. In the group of 1–9-year-old T1D patients with serum MANF concentration over 13.5 ng/ml (*n* = 11) the average levels of IL-1β, IL-2, IL-4, IL-5, IL-10, IL-12 (p70), IL-13, and IFN-γ were 13-56% higher compared to the levels in the patients with lower MANF concentration (*n* = 39, Table 4). However, after Bonferroni correction none of the comparisons reached statistical significance.

## Discussion

This is the first study to report that MANF is detectable in human serum. In healthy controls with average age of 14.6 ± 12.0 years, the mean serum MANF concentration was 7.0 ng/ml. Serum MANF levels were independent of age and gender among the non-diabetic subjects.

As the lack of MANF in mice caused postnatal diabetes^10^, we expected that serum MANF concentrations are decreased in human T1D. In contrast, we found increased serum MANF concentrations in children at the clinical presentation of T1D.

Studies in mice suggest that MANF is involved in the maintenance of ER homeostasis in the β-cells^10^,^11^. As the expression and secretion of MANF has been reported to increase under ER-stress, elevated serum MANF level at the onset of T1D may reflect ER stress in the remaining β-cells, strained with increased demand for insulin production due to reduced β-cell mass^27^ in a chronically hyperglycemic environment, an aspect further impairing insulin secretion and causing increasing ER-stress^28^. An important question is whether the increase in serum MANF, which was observed in the group of 1–9-year-old children but not in older children and adolescents, is related to changes in the functional β-cell mass at the disease onset. At the clinical presentation of T1D, the remaining functional β-cell mass varies greatly from one patient to another, and is positively correlated with age^29^. However, in the current study the average C-peptide levels, an indirect measure of functional β-cell mass, did not differ between the two age groups, suggesting that the circulating MANF levels are not directly related to functional β-cell mass at the diagnosis of T1D.

A subset of 1–9-year-old children with newly diagnosed T1D had very high (>13.5 ng/ml) serum MANF concentrations. The children were younger, they had lower C-peptide levels and their samples were collected closer to the clinical diagnosis of T1D compared to the children with lower (≤13.5 ng/ml) MANF concentrations at the disease presentation. Further studies are needed to clarify, whether extremely high serum MANF concentration is associated with compromised β-cell function at the manifestation of T1D in children.

It is possible that the increased MANF concentrations detected in the 1–9-year-old children with newly diagnosed T1D reflect metabolic decompensation prevailing at the time of diagnosis. However, there were no correlations between blood pH and plasma glucose concentrations measured at diagnosis with MANF concentrations measured 0–22 days from the diagnosis of T1D. Unfortunately we had no data in these patients on glycosylated hemoglobin values at the diagnosis, reflecting the degree of hyperglycemia over the preceding 2–3 months.

Cytokine secretion from β-cells and the infiltrating immune cells is well characterized in the progression to overt T1D^30^. Recombinant MANF has been demonstrated to decrease the expression and secretion of proinflammatory cytokines IL-1β, IL-6 and TNFα *in vitro*^16^,^23^. However, in the analyzed serum samples none of the measured cytokines, including IL-1β, IL-6 and TNFα, differed statistically significantly between the groups of high (>13.5 ng/ml) and lower (≤13.5 ng/ml) serum MANF concentration.

The presence of circulating autoantibodies against β-cell antigens are used as predictive markers for increased risk of disease development in non-diabetic individuals^31^,^32^. MANF serum concentrations were not related to the prevalence of autoantibodies in the patients with T1D or in the autoantibody-positive controls implying that the serum MANF concentration is not related to the autoimmune process in the patients or to the preclinical state of T1D, although compromised β-cell function is present in a subset of non-diabetic individuals with autoantibodies^33^. Furthermore, signs of autoimmunity against MANF were extremely rare in newly diagnosed patients, implying that MANF is not an important autoantigen in T1D.

MANF expression is widespread in mammalian tissues^19^, and whether the observed increase in serum MANF in fact is released from ER-stressed β-cells, remains to be studied. However, the basal level of MANF detected in human serum is not likely to originate from β-cells as MANF levels in the longer-term diabetic patients, where β-cell mass is almost completely destroyed, are comparable to the non-diabetic controls.

Although MANF concentrations remained stable in serum samples after repeated freeze-thaw cycles, the recovery of spiked MANF after one freeze-thaw cycle was significantly lower in the serum of non-diabetic control subjects than in the serum of T1D patients. Thus, it is possible that the stability or detectability of MANF is different in the serum of T1D patients compared to the serum of controls, which, if verified, may give an insight into the mechanism behind the elevated MANF levels detected in children with newly diagnosed T1D.

It is becoming evident that inflammation and ER-stress mediated β-cell failure are involved in the pathogenesis of both T1D and T2D^34^. Obesity was demonstrated to cause ER stress in adipose tissue leading to insulin resistance and activation of inflammatory signaling^35^. Chronic elevation of blood glucose and free fatty acids creates ER stress also in β-cells, leading to β-cell dysfunction and death^36^. Interestingly, MANF-deficiency has recently been linked to T2D. Differently from the insulin-deficient phenotype of MANF knockout mice^10^, a homozygous mutation in the MANF gene was reported in a human patient suffering from T2D and obesity^37^. Thus, it is of interest to study the association of MANF with insulin resistance and type 2 diabetes, as well.

The ability of MANF to specifically enhance the proliferation of adult mouse β-cells^10^ suggests that it could be used as a therapeutic agent either in the preclinical stage or after the diagnosis of diabetes to promote the survival and proliferation of remaining β-cells in humans. As a potential therapeutic protein for diabetes MANF needs further attention and investigation.

## Materials and Methods

### Study population

All study subjects were derived from the Finnish Pediatric Diabetes Register and Sample Repository^38^. Parents or legal guardians of children under 18 years of age participating the study and all study subjects aged 18 years or above gave written informed consent. Children and adolescents aged 10–17 years gave in addition written assent. The study population (Table 1) included 186 children and adolescent with newly diagnosed T1D, and 92 autoantibody-positive and 93 autoantibody-negative controls. In addition, the study population comprised 20 adult patients with longer-term T1D, 20 autoantibody-positive and 20 autoantibody-negative unaffected adults. All the controls were first-degree relatives to a patient affected by T1D. MANF autoantibodies were analyzed from the serum of 94 children with newly diagnosed T1D, 32 autoantibody-positive children, and 91 autoantibody-negative children. For the validation of ELISA, we used blood samples from the Finnish Red Cross (license 5/2013). The methods used in the present study were carried out in accordance with the Declaration of Helsinki. All experimental protocols were approved by the Ethics Committee of the Hospital District of Helsinki and Uusimaa.

### Blood samples

Blood samples were allowed to clot at room temperature (RT) for 10 min, centrifuged at 1,000 g for 10 min and serum was aliquoted and stored at −80 °C.

### MANF ELISA

MaxiSorp (Nunc, Fisher Scientific) 96-well plates were coated overnight at +4 °C with goat anti-human MANF polyclonal antibody (AF3748, R&D Systems) at 1 μg/ml in 50 mmol/l carbonate coating buffer (35 mmol/l sodium bicarbonate, 15 mmol/l sodium carbonate; pH 9.6). The plate was washed once with phosphate buffered saline, 0.05% Tween 20 (PBST), and incubated with blocking buffer (PBST, 1% casein) at RT for 2 h. After washing with PBST, standard samples of recombinant human MANF (P-101-100, Icosagen, Supplementary Methods) ranging from 62.5 to 2,000 pg/ml and serum samples diluted 1:20 in blocking buffer and pre-incubated on ice for 1 h with 500 mg/l IIR, were added to the plate in duplicate and incubated overnight at +4 °C in agitation (100 rpm). The detection antibody, horseradish peroxidase-conjugated mouse anti-human MANF monoclonal antibody (4E12, Icosagen, Supplementary Methods), was incubated on the plate at 1 μg/ml for 5 h in agitation at RT. Washing with PBST was repeated four times before and after the antibody incubation. Antibodies and samples were applied to the plate in 100 μl volume. For detection, 3,3’,5,5’-tetramethylbenzidine was used according to the manufacturer’s instructions (DuoSet ELISA Development System, R&D Systems). The absorbance was read using a plate reader (VICTOR^3^, Perkin Elmer) at 450 nm and 540 nm (for wavelength correction).

### Validation of MANF ELISA

The specificity, sensitivity, dynamic range, accuracy (% Relative Error, % RE = derived concentration/expected concentration x 100%) and precision (% Coefficient of Variation, % CV = SD/mean x 100%) of the assay were determined according to recommendations^39^. Specificity of the ELISA was tested with recombinant human CDNF (500 ng/ml; Icosagen) and mouse tissue lysates (1.4–2.0 mg/ml of protein). Sensitivity was determined as the mean absorbance value of ten zero samples added by three SDs and calculating resulting MANF concentration from the standard curve. The dynamic range was determined by the accuracy of the back-calculated concentration values for each standard curve point from six individual assays. A mean accuracy of ±15% RE and precision of ≤15% CV was considered acceptable. Intra-assay precision was determined by measuring three samples with varying MANF concentration in replicates of ten on different parts of a plate. Interassay precision was determined by running three different samples in duplicate on six independent assays on different days. Precision of ≤20% CV was considered acceptable.

### Detection and blocking of heterophilic antibodies

The control ELISA, constructed for the evaluation of possible interference by serum heterophilic antibodies, was run exactly as the MANF ELISA except that the coating antibody was goat anti-human CDNF (AF5097, R&D Systems). We decided to use IIR to block the heterophilic antibody-interference based on previous positive reports^40^,^41^. Activity of IIR was tested with diluted (1:20) serum samples at final concentrations of 100 mg/l and 500 mg/l. Linearity of dilution and recovery of spiked recombinant human MANF in serum was tested in the presence of 500 mg/l IIR. Recovery of spiked recombinant human MANF (250 and 500 pg/ml) was calculated after subtracting the endogenous MANF concentration from the results. For assessing linearity of dilution, recovery of endogenous MANF after serial dilutions (1:40; 1:80; 1:160) was calculated in relation to the 1:20 dilution. Recoveries of 80–120% were considered acceptable.

### Stability of MANF in serum

Stability of endogenous and spiked MANF in serum under repeated freeze-thaw cycles was tested in serum samples from T1D patients and autoantibody-negative controls (*n* = 4 for both). The sera were aliquoted, and parallel samples were spiked with recombinant human MANF (á 500 pg/ml). The sera were exposed to 1, 2, 4 or 8 freeze-thaw cycles before analysis on ELISA. Samples were thawed on ice and refrozen at −80 °C. Endogenous MANF concentration in the sera from T1D patients was 5.8, 9.5, 12.1, and 18.3 ng/ml, and in the sera from autoantibody-negative controls 4.1, 9.0, 11.2, and 14.8 ng/ml. Recovery of spiked MANF was analyzed by subtracting the endogenous MANF concentration analyzed in the parallel sample.

### C-peptide levels

Random serum C-peptide concentrations were measured by Cobas e 411 analyzer (Roche Diagnostics). We have previously shown that there is a strong correlation between random serum C-peptide levels and serum C-peptide concentrations measured 120 min after a standardized meal and the 24-h urinary C-peptide secretion^42^.

### Radiobinding assays

We analyzed five different diabetes-associated autoantibodies, i.e. ICA, IAA, autoantibodies to GADA, IA-2A, and ZnT8A as described^43^. Antibodies against MANF were analyzed with a radiobinding assay^44^. Human MANF complementary DNA (cDNA) in pCR3.1 vector was used to produce ^35^S-labeled MANF by *in vitro* translation using TNT Coupled Reticulocyte Lysate System (Promega). Briefly, 5 μl of serum was incubated at +4 °C overnight with 10,000 cpm of labeled MANF diluted in 50 μl of TBST buffer [50 mmol/l Tris-HCl (pH 7.4), 150 mmol/l NaCl, 0.1% Tween-20]. Immune complexes were precipitated with Protein A-Sepharose. After washing, bound radioactivity was counted by a liquid scintillation counter (1450 MicroBeta Trilux, Perkin Elmer).

### Cytokine levels

IL-1β, IL-2, IL-4, IL-5, IL-6, IL-10, IL-12 (p70), IL-13, IFN-γ and TNF-α levels were analyzed from 1:4 diluted sera by Bio-Plex Precision Pro Human Cytokine Assay (Bio-Rad).

### Statistical analysis

Differences in the stability of MANF in serum after freeze-thaw cycles were analyzed by repeated measures ANOVA and independent samples t-test. Serum MANF, C-peptide and cytokine concentrations showed a non-normal distribution (Shapiro-Wilk test, *p* < 0.01) in the populations studied; thus non-parametric statistical tests were applied. Differences between two groups were analyzed with Mann-Whitney U test and between three or more groups with Kruskall-Wallis H test in adjunct with Tukey HSD post-hoc test of the rank-ordered data. Correlations were analyzed with the non-parametric Spearman’s test (r_s_). A *p* value <0.05 was considered to indicate statistical significance. Unless otherwise indicated, all results are expressed as average ± SD (median). Statistical analyses were performed using Software Package for Social Science (SPSS) v. 21.0 (Chicago, IL).

## Additional Information

**How to cite this article**: Galli, E. *et al*. Increased circulating concentrations of mesencephalic astrocyte-derived neurotrophic factor in children with type 1 diabetes. *Sci. Rep.* **6**, 29058; doi: 10.1038/srep29058 (2016).

## Supplementary Material

Supplementary Information

## Figures and Tables

**Table 1:** Study groups.

	T1D	AAB+	AAB−
Age 1–9 years			
* n*	98	48	48
* *Males, *n* (%)	53 (54.1%)	24 (50.0%)	23 (47.9%)
* *Age, years (mean ± SD)	6.0 ± 2.6	6.3 ±2.4	6.2 ±2.3
* *Age, years (min-max)	1.1–9.9	0.9–9.6	1.0–9.7
* *Duration of T1D, days (mean ± SD)	5.2 ±3.5	—	—
* *Duration of T1D, days (min-max)	0–22	—	—
Age 10–17 years			
* n*	88	44	45
* *Males, *n* (%)	50 (56.8%)	27 (61.4%)	28 (62.2%)
* *Age, years (mean ± SD)	13.2 ±1.6	13.1 ±1.8	13.1 ±1.8
* *Age, years (min-max)	10.0–16.8	10.2–17.0	10.0–16.9
* *Duration of T1D, days (mean ± SD)	5.8 ±5.2	—	—
* *Duration of T1D, days (min-max)	0–24	—	—
Age 25–52 years			
* n*	20	20	20
* *Males, *n* (%)	10 (50%)	10 (50%)	10 (50%)
* *Age, years (mean ± SD)	38.3 ±7.3	38.5 ±7.9	38.3 ±7.7
* *Age, years (min-max)	27.3–52.0	25.2–52.5	25.4–50.9
* *Duration of T1D, years (mean ± SD)	20.7 ±12.0	—	—
* *Duration of T1D, years (min-max)	3.8–40.3		

T1D = Type 1 diabetes patients, AAB+ = autoantibody-positive non-diabetic controls, AAB− = autoantibody-negative non-diabetic controls.

**Table 2:** MANF serum concentrations (ng/ml) in the study groups.

	T1D	AAB+	AAB−
Age 1–9 years			
* n*	98	48	48
* *Mean ± SD	11.1 ±7.5	7.5 ±3.8	7.2 ±2.8
* *Median; 25%-75%	9.5; 6.2–12.9	6.1; 5.2–9.0	7.0; 5.4–8.3
* *Min-Max	2.3–40.1	2.2–22.8	2.9–14.9
Age 10–17 years			
* n*	88	44	45
* *Mean ± SD	8.1 ±4.1	6.8 ±3.2	7.1 ±3.7
* *Median; 25%-75%	7.2; 5.4–9.7	6.0; 4.2–8.7	6.6; 5.0–8.7
* *Min-Max	2.7–19.6	2.3–14.5	1.4–21.8
Age 25–52 years			
* n*	20	20	20
* *Mean ± SD	6.5 ±2.9	7.4 ±4.2	6.0 ±2.7
* *Median; 25%-75%	6.3; 4.3–7.2	6.5; 5.5–7.7	5.6; 3.9–8.0
* *Min-Max	3.1–13.7	2.3–22.3	2.7–12.3

T1D = Type 1 diabetes patients, AAB+ = autoantibody-positive non-diabetic controls, AAB− = autoantibody-negative non-diabetic controls.

**Table 3:** Serum C-peptide and MANF concentrations in the study groups.

	Study group	*n*	C-peptide (nmol/l)	MANF (ng/ml)
**Age** 1–9 **years**	T1D	84	0.27 ± 0.22	11.2 ± 7.8
	AAB+	36	0.63 ± 0.32	7.5 ± 3.9
	AAB−	35	0.97 ± 0.61	6.9 ± 2.6
**Age** 10–17 **years**	T1D	79	0.33 ± 0.31	7.7 ± 3.7
	AAB+	37	0.93 ± 0.49	6.7 ± 3.2
	AAB−	38	0.92 ± 0.44	7.0 ± 3.8
**Age** 25–52 **years**	T1D	20	0.08 ± 0.15	6.5 ± 2.9
	AAB+	20	1.09 ± 0.62	7.4 ± 4.2
	AAB−	20	0.99 ± 0.43	6.0 ± 2.7

Data is presented as an average ± SD. T1D = Type 1 diabetes patients, AAB+ = autoantibody-positive non-diabetic controls, AAB− = autoantibody-negative non-diabetic controls.

**Table 4:** Average (±SD) concentrations of circulating cytokines in 1–9-year-old T1D patients with extremely high (>13.5 ng/ml) and lower (≤13.5 ng/ml) MANF concentrations.

	Serum MANF (ng/ml)	Cytokine (pg/ml)	Difference (%)	*p*-value
IL-1β	≤13.5	2.2 ± 1.1		
	>13.5	2.7 ± 0.9	+22.7	0.089
IL-2	≤13.5	14.6 ± 8.1		
	>13.5	19.4 ± 8.3	+32.9	0.066
IL-4	≤13.5	4.8 ± 2.1		
	>13.5	6.0 ± 2.0	+25.0	0.057
IL-5	≤13.5	13.6 ± 18.0		
	>13.5	15.3 ± 8.0	+12.5	0.11
IL-6	≤13.5	112.5 ± 187.4		
	>13.5	55.4 ± 53.2	−50.8	0.84
IL-10	≤13.5	21.5 ± 14.3		
	>13.5	33.6 ± 23.0	+56.3	0.015
IL-12	≤13.5	5.6 ± 2.7		
	>13.5	7.1 ± 2.2	+26.8	0.039
IL-13	≤13.5	3.6 ± 1.6		
	>13.5	4.6 ± 1.5	+27.8	0.045
INF-γ	≤13.5	21.7 ± 11.1		
	>13.5	29.2 ± 14.2	+34.6	0.066
TNF-α	≤13.5	2.8 ± 1.5		
	>13.5	2.8 ± 0.9	0.0	0.32

The difference (%) in cytokine concentration between the two groups was calculated as compared to ≤13.5 ng/ml group. The Bonferroni corrected *p*-value of the repeated Mann-Whitney U test is 0.005 (=0.05/10). *n* = 39 for MANF ≤13.5 ng/ml, *n* = 11 for MANF >13.5 ng/ml.
