# Supplementary material for: Increased circulating concentrations of mesencephalic astrocyte-derived neurotrophic factor in children with type 1 diabetes
Source: Sci Rep. 2016 Jun 30;6:29058. doi: 10.1038/srep29058 (PMC4928177; doi:10.1038/srep29058)
Supplement: Supplementary Information [file srep29058-s1.pdf]

Increased circulating concentrations of mesencephalic astrocyte-derived neurotrophic factor in children with type 1 diabetes

Emilia Galli[1,2], Taina Härkönen[3,4], Markus T. Sainio[1], Mart Ustav[5], Urve Toots[5], Arto Urtti[2], Marjo Yliperttula[2], Maria Lindahl[1], Mikael Knip[3,4,6], Mart Saarma[1,*], and Päivi Lindholm[1,*]

[1]Institute of Biotechnology, University of Helsinki, Finland

[2]Division of Pharmaceutical Biosciences, Centre for Drug Research, University of Helsinki, Finland

[3]Children's Hospital, University of Helsinki and Helsinki University Central Hospital, Finland

[4]Research Programs Unit, Diabetes and Obesity, University of Helsinki, Finland

[5]Icosagen Ltd., Tartu, Estonia

[6]Folkhälsan Research Center, Helsinki, Finland

*These authors contributed equally to this work

Correspondence and requests for materials should be addressed to P.L. (email: paivi.pulkkila@helsinki.fi)

Supplementary Methods

Production of recombinant human MANF. Quattromed Cell Factory (QMCF) technology was used for recombinant human MANF production. The core of the QMCF Technology has been described previously [1]. Codon optimized human MANF cDNA was cloned into pQMCF-1 expression vector, purified by a plasmid mini endofree kit (Favorgen) and transfected by electroporation (230 V, 975 µF) into CHO-based cell line CHOEBNALT85. Transfected cells were grown in shaker flasks (Kühner) at 110 rpm, in 8% CO_2 at 37°C in chemically defined serum free media. To select plasmid-containing cells, G418 (700 μg/ml) was added 48 h after transfection. For production, temperature was lowered to 30°C and the cell culture (200 ml) was fed by media supplement (Life Technologies). After incubation, cells were removed by centrifugation at 1,000g for 15 min at 15°C and the supernatant containing recombinant human MANF was stored at -20°C. MANF was purified by ion-exchange chromatography and gel-filtration. Purified recombinant human MANF was analyzed by sodium dodecyl sulphate-polyacrylamide gel electrophoresis (SDS-PAGE) followed by Coomassie-staining and mass spectrometry, and was more than 98% pure.

Monoclonal anti-human MANF antibody. Recombinant human MANF was used as an antigen for eliciting an immune response in 4-week-old female Balb/c mice. Booster injections were given on the third, sixth and ninth week after the first injection. Three days from the last injection spleen cells were isolated, fused with myeloma Sp2/O cells using polyethylene glycol 4000, and resuspended in RPMI 1640, 10% fetal calf serum (FCS), supplemented with the selecting agent hypoxanthine-aminopterin-thymidine. Ten to 14 days after the fusion supernatants of the hybridoma clones were screened for MANF reactivity by indirect ELISA and positive hybridoma clones were subcloned. Subclass of the selected monoclonal antibody was defined by ELISA using subclass specific horseradish peroxidase (HRP)-conjugated anti-mouse IgG antibodies. For antibody production, the hybridoma cells were suspended into cell compartment media (RPMI 1640, 10%

FCS, 25 mmol/l HEPES, L-glutamine). After incubation, the cell suspension was centrifuged at 215g for 10 min at room temperature. The antibody was purified from the supernatant by Protein G affinity chromatography and gel-filtrated into PBS. The monoclonal antibody was conjugated to HRP (Pierce, Thermo Scientific) by simplified sodium periodate method according to Tijssen et al. [2].

Supplementary References

1. Silla, T. *et al*. Episomal maintenance of plasmids with hybrid origins in mouse cells. *J. Virol.* **79**, 15277-15288 (2005).

2. Tijssen, P. in *Practice And Theory Of Enzyme Immunoassays: Laboratory Techniques in Biochemistry and Molecular Biology* (eds Burdon, R. H., van Knippenberg, P.H.) 236-240 (Elsevier, 1988).

Supplementary Tables

Recombinant human MANF (pg/ml)	62.5	125.0	250.0	500.0	1 000.0	2 000.0
Mean recovery	61.03	119.29	269.00	521.78	1 017.00	1 888.08
Mean % RE	99.2	95.7	106.0	103.4	101.9	95.3
Mean % CV	6.6	6.2	3.4	4.1	2.2	3.6

Supplementary Table S1. Dynamic range of the MANF ELISA. For each standard point, the mean accuracy and precision values of six runs are shown. The mean accuracy and precision were within 10% RE and 10% CV, respectively. The individual accuracy values were within 15% RE of the nominal values, and precision values were within 15% CV. % RE = % relative error, % CV = % coefficient of variation.

Concentration of recombinant human MANF	Intra-assay precision		Interassay precision	
	Mean (pg/ml)	% CV	Mean (pg/ml)	% CV
Low	120.6	8.4	173.4	6.7
Medium	338.8	4.4	586.7	3.1
High	1112.7	11.5	1638.8	6.8

Supplementary Table S2. Intra-assay precision (repeatability) and inter-assay precision (reproducibility) of the MANF ELISA. Results are shown as a mean of ten replicates for the intra-assay precision, and as a mean of six separate assays for the inter-assay precision, respectively. % CV = % coefficient of variation.

Sample	Average OD (n=2)	SD
Recombinant human CDNF (500 ng/ml)	0.002	0.001
Mouse heart lysate	-0.008	0.006
Mouse testis lysate	-0.012	0.003
Recombinant human MANF (pg/ml)		
2000	0.492	0.027
1000	0.266	0.012
500	0.137	0.010
250	0.071	0.008
125	0.032	0.003
62.5	0.016	0.001
0	0.000	0.001

Supplementary Table S3. Specificity of the MANF ELISA. For 500 ng/ml recombinant human CDNF, the assay gave OD-value below the average OD value of the lowest limit of quantitation (i.e. 62.5 pg/ml), and the calculated concentration value was 7.3 pg/ml which is out of the standard range and below sensitivity of the assay (i.e. 45 pg/ml). For mouse heart and testis lysates diluted 1:5 in blocking buffer, the dilution containing 2.0 and 1.4 mg/ml total protein, respectively, the assay gave OD-values below the blank sample. OD = optical density.

	Linearity of dilution (%)			Spike & recovery (%)	
	Dilution factor			Spike (pg/ml)	
**Sample**	**1:40**	**1:80**	**1:160**	**250**	**500**
Serum 1	104.7	105.6	115.5	105.8	100.4
Serum 2	96.0	105.9	108.8	98.1	109.6
Serum 3	106.3	106.3	111.2	102.4	93.5
Mean recovery (%)	102.3	105.9	111.8	102.1	101.2

Supplementary Table S4. Linearity of dilution (%) and recovery (%) of spiked recombinant human MANF in the presence of 500 mg/l IIR. For assessing linearity of dilution, recovery of endogenous MANF after serial dilutions (1:40; 1:80; 1:160) was calculated in relation to the 1:20 dilution. Recovery of spiked recombinant human MANF, in serum samples diluted 1:20, was calculated after subtracting the endogenous MANF concentration from the results.

T1D samples with high MANF conc. result	No.	MANF (ng/ml)	MANF ELISA (OD)	Control ELISA (OD)	Background (% of MANF ELISA OD)
	1	40.1	0.525	0.004	0.8
	2	39.1	0.503	-0.010	-
	3	37.0	0.486	-0.001	-
	4	31.4	0.476	0.003	0.6
	5	31.0	0.474	0.005	1.1
	6	28.3	0.435	0.008	1.8
	7	22.8	0.287	0.072	25.0
	8	22.1	0.287	-0.017	-
	9	21.8	0.246	0.135	54.8
	10	21.2	0.249	-0.019	-
	11	20.0	0.248	0.028	11.3
	12	19.5	0.243	0.013	5.5
	13	19.1	0.242	0.004	1.6
	14	18.8	0.238	0.007	3.0
	15	18.3	0.237	0.007	2.9
	16	17.9	0.241	0.003	1.1
	17	17.5	0.243	0.019	8.7
	18	17.1	0.229	0.008	3.6
	19	17.1	0.233	0.010	4.2
	20	16.7	0.223	0.006	2.5
	21	16.2	0.227	0.003	1.5
**AAB- controls**	**No.**	**MANF (ng/ml)**	**MANF ELISA (OD)**	**Control ELISA (OD)**	**Background (% of MANF ELISA OD)**
	22	12.6	0.167	-0.002	-
	23	12.3	0.163	-0.005	-
	24	11.2	0.148	-0.004	-
	25	10.5	0.139	-0.004	-
	26	8.0	0.106	0.001	1
	27	7.2	0.095	0.008	8.5
	28	7.0	0.096	0.000	-
	29	6.2	0.089	0.010	11.2
	30	6.1	0.082	0.001	0.8
	31	5.8	0.093	0.038	40.8
	32	4.8	0.063	0.001	2.2
	33	4.6	0.069	-0.004	-
	34	4.1	0.051	0.005	8.9
	35	3.9	0.053	0.002	2.9

Supplementary Table S5. Remaining background in selected study samples. Selected serum samples from the study population were analyzed in parallel with MANF ELISA and control ELISA for remaining background in the presence of IIR (500 mg/l). Presented MANF concentration values were measured previously from the same samples and were used in the data analysis. T1D = Type 1 diabetes patients, AAB- = autoantibody-negative controls, OD = optical density.

**1-9-year-olds**										
**Group**	**T1D**					**Non-diabetic, AAB+**				
**Occurrence of the AAB**	**Yes**		**No**			**Yes**		**No**		
**AAB**	*n*	MANF (ng/ml)	*n*	MANF (ng/ml)	*p* value	*n*	MANF (ng/ml)	*n*	MANF (ng/ml)	*p* value
ICA	87	11.0±7.4	8	11.8±8.5	0.72	43	7.0±2.6	5	11.7±8.8	0.47
IAA	43	11.2±7.4	52	10.9±7.6	0.49	22	7.7±4.5	26	7.4±3.3	0.58
GADA	58	11.0±6.6	37	11.1±8.7	0.44	38	7.5±4.0	10	7.5±3.2	0.93
IA2A	66	11.5±8.5	29	9.9±4.3	0.97	33	7.4±3.2	15	7.7±5.1	0.75
ZnT8A	32	12.8±9.3	19	11.9±8.0	0.87	29	7.5±3.1	19	7.5±4.9	0.51
**10-17-year-olds**										
**Group**	**T1D**					**Non-diabetic, AAB+**				
**Occurrence of the AAB**	**Yes**		**No**			**Yes**		**No**		
**AAB**	*n*	MANF (ng/ml)	*n*	MANF (ng/ml)	*p* value	*n*	MANF (ng/ml)	*n*	MANF (ng/ml)	*p* value
ICA	63	8.0±3.9	12	9.7±4.4	0.23	39	6.8±3.2	5	6.9±3.0	0.75
IAA	20	8.1±3.2	55	8.3±4.3	0.59	16	6.8±3.5	28	6.8±3.0	0.96
GADA	52	8.1±3.9	23	8.6±4.2	0.68	39	6.6±3.2	5	8.4±2.6	0.11
IA2A	53	7.7±3.4	22	9.6±5.1	0.27	23	7.4±3.5	21	6.1±2.7	0.23
ZnT8A	30	8.6±4.8	6	10.4±5.8	0.31	22	6.3±2.6	22	7.3±3.7	0.48
**25-52-year-olds**										
**Group**	**T1D**					**Non-diabetic, AAB+**				
**Occurrence of the AAB**	**Yes**		**No**			**Yes**		**No**		
**AAB**	*n*	MANF (ng/ml)	*n*	MANF (ng/ml)	*p* value	*n*	MANF (ng/ml)	*n*	MANF (ng/ml)	*p* value
ICA	9	6.7±3.4	11	6.4±2.6	0.97	17	7.6±4.5	3	6.2±0.7	0.63
IAA	19	6.1±2.5	1	13.7	0.10	2	4.2±2.7	18	7.8±4.2	0.21
GADA	11	6.3±2.9	9	6.8±3.1	0.70	13	6.7±2.3	7	8.8±6.4	0.50
IA2A	9	6.5±2.5	11	6.5±3.4	0.57	11	8.6±5.0	9	6.0±2.5	0.34
ZnT8A						7	8.8±6.1	13	6.6±2.8	0.45

Supplementary Table S6. **Average (± SD) concentrations of circulating MANF in relation to the occurrence of T1D-predictive autoantibodies in patients and in autoantibody-positive non-diabetic siblings.** The Bonferroni corrected *p* value in the repeated Mann-Whitney U test is 0.01 (= 0.05/5). T1D = Type 1 diabetes patients, AAB+ = autoantibody-positive non-diabetic controls.

Supplementary Figures

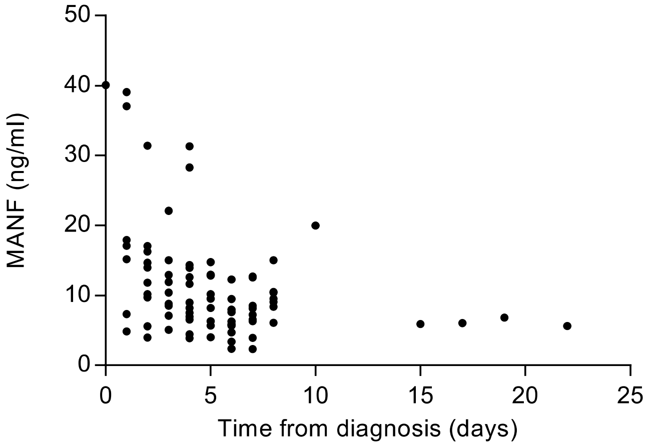

Figure S1. Inverse correlation of MANF and the time from diagnosis to sample collection (r_s=-0.38, $p<0.001$, n=84) in the 1-9-year-old children with T1D, whose C-peptide levels were measured.

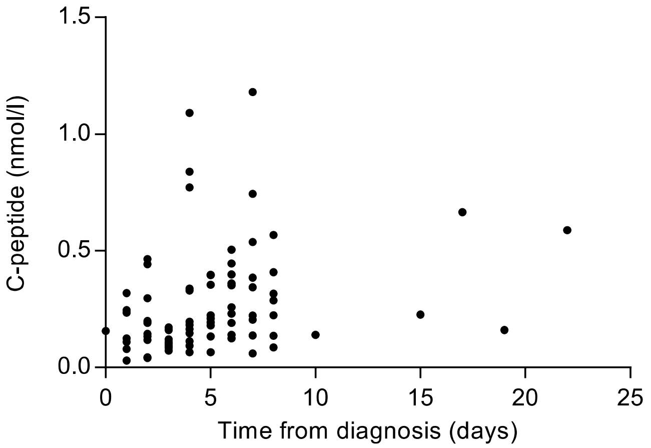

Figure S2. Positive correlation of C-peptide and the time from diagnosis to sample collection (r_s=0.36, p=0.001, n=84) in the 1-9-year-old children with T1D.
